# COVID-19-Associated Spontaneous Pneumomediastinum and Pneumopericardium: Review of Case Series

**DOI:** 10.7759/cureus.19546

**Published:** 2021-11-13

**Authors:** Krithika Suresh, Michael W Figart, Talha Mehmood, Asfandyar Butt, Amanpreet Sherwal

**Affiliations:** 1 Internal Medicine, Conemaugh Memorial Medical Center, Johnstown, USA; 2 Cardiothoracic Surgery, Conemaugh Memorial Medical Center, Johnstown, USA

**Keywords:** chest tube, mechanical ventilation, covid-19, pneumopericardium, pneumomediastinum

## Abstract

Coronavirus disease 2019 (COVID-19) and its spectrum of respiratory illnesses ranging from mild to severe and critically ill have been well established. Spontaneous pneumomediastinum and pneumopericardium (PP) appear to be less reported entities and have been found to be reported complications in COVID-19 infection. Pneumomediastinum (PM) and PP are characterized by the presence of air in the mediastinal and pericardial cavity, respectively. Although, generally, secondary to trauma or underlying lung conditions like asthma, bronchiolitis obliterans, and blunt trauma, it can also occur spontaneously without an evident primary cause. PM and PP are increasingly reported complications in COVID-19 patients adversely affecting clinical outcomes. We present a case series of patients with spontaneous pneumomediastinum and pneumopericardium in the presence of underlying COVID-19 infection and their management at our academic medical center.

## Introduction

An outbreak of coronavirus disease 2019 (COVID-19) caused by severe acute respiratory syndrome coronavirus 2 (SARS-CoV-2) during December 2019, initially reported in Wuhan, China, spread around the world and was declared a pandemic [1]. The initial clinical case series from China largely comprised hospitalized patients with severe pneumonia. Data mainly suggested that about 80% of patients have mild disease, 20% require hospital admission, and about 5% require intensive care admission [2].

Most of the intensive care admissions develop COVID-19-associated acute respiratory distress syndrome (ARDS) often requiring mechanical ventilation and the mortality rates are often higher among these patients owing to multi-organ failure. Mortality ranged from 40.8% among patients receiving only invasive mechanical ventilation, 39% for those receiving extracorporeal membrane oxygenation (ECMO) to 71.6% for those receiving invasive mechanical ventilation, vasoactive drugs, and new renal replacement therapy [3]. Spontaneous pneumomediastinum (SPM) and pneumopericardium (PP) are increasingly reported complications among COVID-19 patients, occurring in the absence of underlying predisposing lung conditions or trauma.

## Case presentation

Case 1

**Figure 1:**
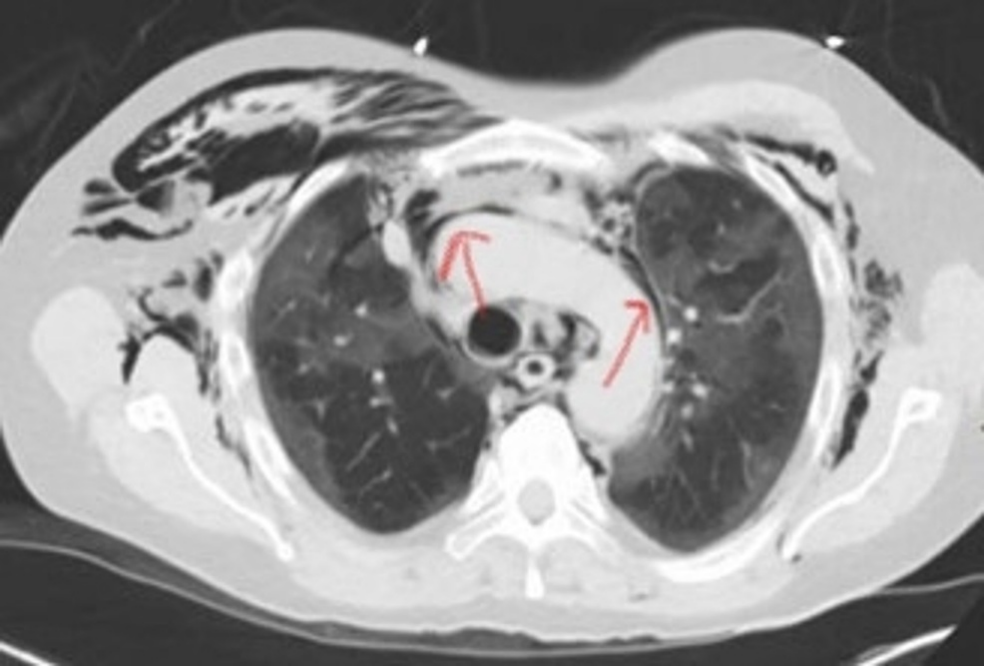
CT chest showing large pneumomediastinum, pneumopericardium, and extensive chest wall and bilateral neck base subcutaneous emphysema

A 66-year-old male with a past medical history (PMH) significant for hypertension, chronic kidney disease (CKD) stage III, and migraine presented to the emergency department (ED) with cough and shortness of breath (SOB). He was maintaining oxygen saturation (SpO_2_) at 87% on room air and vitals were stable. He also reported a change in taste and smell. His COVID-19 RNA polymerase chain reaction (PCR) test was positive. His initial chest x-ray showed bilateral interstitial and ground-glass opacities. He was initially admitted to the medical floor and started on dexamethasone 6 mg IV daily and remdesivir 200 mg IV on day one followed by 100 mg IV daily for four days. Ceftriaxone 1 g IV daily and azithromycin 500 mg IV daily were added for super-imposed bacterial pneumonia. He was noted to have worsening hypoxemia necessitating oxygen supplementation via a high flow nasal cannula at 50% fraction of inspired oxygen (FiO_2_) at 15 liter/minute (L/min) after which his SpO_2_ improved to 90%. Diuresis was achieved by giving bumetanide 2 mg IV as needed. Echocardiography showed a normal ejection fraction (EF) of 55-60%, and although there was no element of heart failure in order to achieve net negative fluid balance, diuresis was administered as needed. He was also given a trial of continuous positive airway pressure (CPAP) after which his clinical status improved briefly. He was placed on CPAP intermittently with pressure support of 7 cm H_2_O and heated high flow nasal cannula (HFNC) at 50-60% FiO_2_ in order to maintain SpO_2_ above 88%. He was also recommended to be self-prone in order to further improve his oxygenation above 90%. On day 11 of hospitalization, he deteriorated and his oxygen requirements increased from 50% FiO_2_ at 15 L/min to FiO_2_ of 80% at 50 L/min on a high flow nasal cannula. At this time, he was transferred to the ICU for further management. He was given a dose of tocilizumab 8 mg/kg once. CT chest was obtained which showed bilateral ground-glass opacities, large pneumomediastinum, pneumopericardium, and extensive chest wall, and bilateral neck base subcutaneous emphysema (Figure 1).

An x-ray esophagogram was done to rule out esophageal perforation and was negative. We tried to avoid positive pressure ventilation with CPAP/bilevel positive airway pressure (BiPAP) and supplemental oxygen via high flow nasal cannula was continued with goal oxygen saturations above 86% necessitating maximal settings of 100% FiO_2_ at 60 L/min. Broad-spectrum antibiotic coverage with meropenem and antifungal coverage with micafungin were initiated for suspected esophageal perforation due to the patient’s clinical deterioration and were later discontinued when x-ray esophagogram was negative for esophageal pathology. He was started on inhaled epoprostenol at 8 mL/h initially with a 20,000 ng/mL solution and later titrated down to 10,000 ng/mL solution. His oxygenation continued to worsen with SpO_2 _dropping to less than 80%, and cardiothoracic (CT) surgery was consulted for extracorporeal membrane oxygenation (ECMO) cannulation. He was started on ECMO on day 13 of hospitalization. His oxygenation did improve while on ECMO. On day 15, his respiratory status acutely decompensated requiring intubation. He was then transferred to a higher center for further management.

Case 2

**Figure 2:**
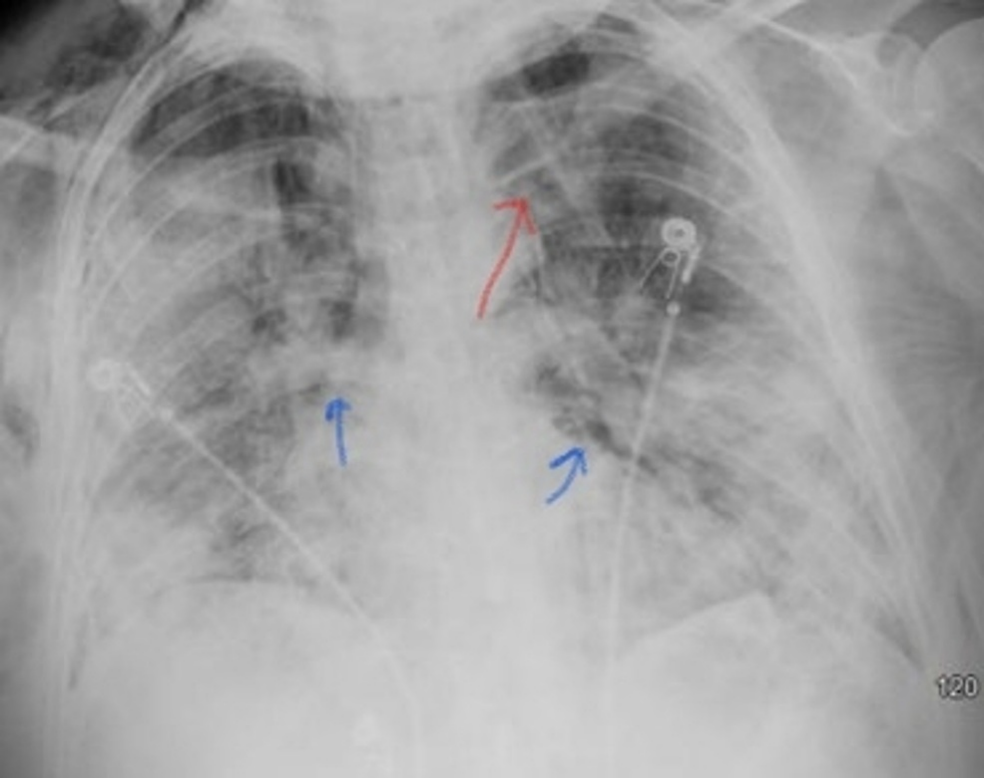
X-ray chest showing marked subcutaneous emphysema, pneumomediastinum (blue arrows), and small left apical pneumothorax (red arrow)

A 47-year-old female with a PMH significant for systemic lupus erythematosus (SLE) on prednisone, Sjogren’s syndrome, Hashimoto’s thyroiditis, and gastroesophageal reflux disease (GERD) presented to the ED with worsening SOB, nausea, and vomiting. In the ED, she was tachycardic with a heart rate (HR) of 102 beats/minute, respiratory rate (RR) of 26 breaths/min, temperature of 39.1 °C, and she was hypoxemic with SpO_2_ of 87% on room air and placed on 3 L oxygen via nasal cannula. Initial CT pulmonary embolism (CT PE) study showed bilateral upper and lower lobe infiltrates. She was given a dose of ceftriaxone 2 g IV and azithromycin 500 mg IV and transferred to the medical floor for further management. Her SARS-CoV-2 PCR RNA came back positive the following day. She was treated with a total five-day course of remdesivir with 200 mg IV on day one followed by 100 mg IV daily for four more days, a 10-day course of dexamethasone 6 mg IV daily, and seven days of ceftriaxone 1 g IV daily, and azithromycin 500 mg IV daily for superimposed bacterial pneumonia. Her oxygenation continued to worsen, and she remained hypoxic with saturation in the low 80s and was placed on 15 L non-rebreather (NRB). She also became increasingly agitated and hypoxemic and was transitioned to high flow nasal cannula (HFNC) initially at 60% FiO_2_ increasing to a maximum setting of 90% FiO_2_ at 60 L/min. X-ray chest showed marked subcutaneous emphysema not previously visualized, pneumomediastinum, and small left apical pneumothorax (Figure 2).

**Figure 3:**
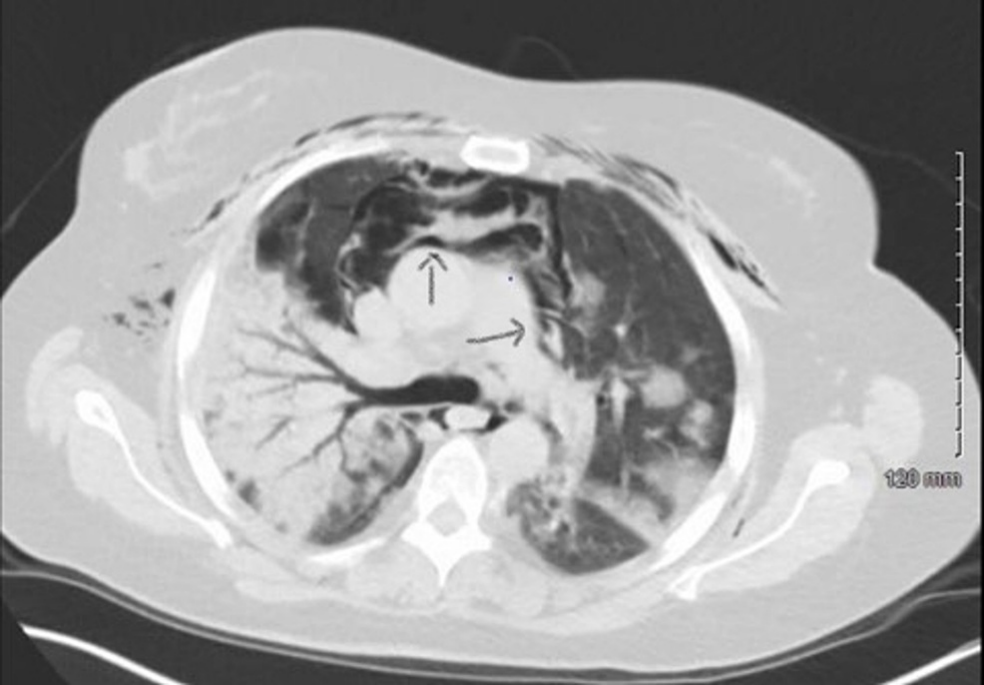
CT chest showing severe diffuse bilateral pneumonia, extensive pneumomediastinum, pneumopericardium, and subcutaneous emphysema

As she clinically deteriorated, she was transferred to the ICU on day 11 of hospitalization for further management. CT chest without contrast was done and it showed severe diffuse bilateral pneumonia, extensive pneumomediastinum, pneumopericardium, and subcutaneous emphysema (Figure 3).

Cardiothoracic surgery was consulted, and a right chest tube was placed. Diuresis was achieved with a furosemide infusion at 5 mg/h and titrated up to 10 mg/h to achieve optimal response. She was treated with broad-spectrum antibiotics vancomycin 20 mg/kg/dose every 12 hours with monitoring of trough levels and piperacillin-tazobactam 4.5 g every six hours due to clinical deterioration. She was also on a heparin drip per venous thromboembolism (VTE) protocol for anticoagulation. Non-invasive ventilation was avoided due to these findings; however, as she continued to worsen with SpO_2_ below 80%, she required intubation and mechanical ventilation. She was started on chemical paralysis with cisatracurium 0.2 mg/kg loading dose followed by a maintenance infusion rate of 2 mcg/kg/min to improve her oxygenation. She was also placed in a prone position multiple times to improve her acute respiratory distress syndrome (ARDS). Prone position protocol involved the patient being in the prone position for 18 hours and then supine for six hours. Her peak pressures were less than 35 cm H_2_O with plateau pressure <30 cm H_2_O and it improved her SpO_2_ to >88%. Repeat CT chest showed improvement of her pneumothorax and pneumomediastinum. However, her course was complicated by heparin-induced thrombocytopenia (HIT) for which anticoagulation was transitioned to argatroban 1 mcg/kg/min with dose titrated based on activated partial thromboplastin time (aPTT). Given her continued clinical deterioration, the patient's family decided to transition care to comfort, and eventually, the patient passed away on day 25 of hospitalization.

Case 3

**Figure 4:**
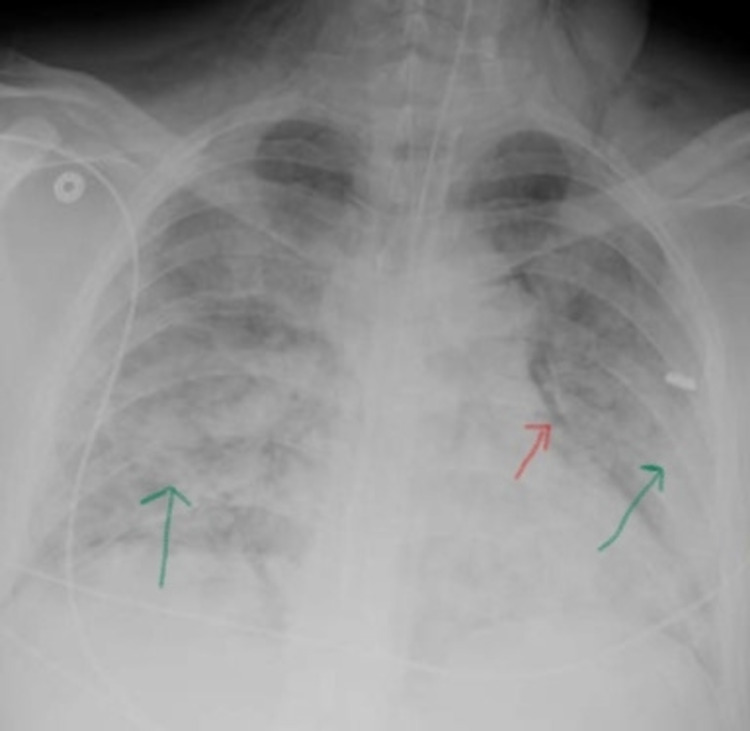
X-ray chest showing pneumomediastinum (red arrow) and bilateral extensive airspace opacifications (green arrows)

A 33-year-old male with a PMH significant for asthma presented initially to an outside hospital (OSH) for worsening SOB and was diagnosed with COVID-19 pneumonia. While at the OSH, his initial oxygen saturation was 88% necessitating supplemental oxygen via nasal cannula. He was admitted on the floor where he was treated with a course of remdesivir 200 mg IV on day one followed by 100 mg daily for four days and methylprednisolone 40 mg every eight hours. He was also treated with azithromycin 500 mg IV daily and ceftriaxone 1 g IV daily for superimposed bacterial pneumonia. His oxygenation continued to worsen necessitating a HFNC. On day eight of hospitalization, he decompensated with tachypnea and oxygen saturation of 84% on 15 L non-rebreather, was intubated, and then transferred to our facility the following day. CT chest obtained at OSH before transfer showed pneumomediastinum and extensive subcutaneous emphysema and was unable to exclude pulmonary embolism. On arrival to our facility, an x-ray chest was done which confirmed pneumomediastinum and showed bilateral extensive airspace opacifications (Figure 4).

Due to worsening ARDS, he was placed on cisatracurium infusion 0.2 mg/kg loading dose followed by a maintenance infusion rate of 2 mcg/kg/min to achieve chemical paralysis. Diuresis was achieved with furosemide infusion initially at 5 mg/h and titrated up to 10 mg/h to achieve optimal diuresis. He was given a dose of tocilizumab 8 mg/kg and started on dexamethasone 6 mg IV daily. Heparin infusion per VTE protocol was started for anticoagulation. An echocardiogram was obtained which showed no evidence of right heart strain with a normal EF of 55-60%. CT surgery was consulted and recommended no acute surgical intervention. He was placed in a prone position on day 10 of hospitalization due to a worsening arterial oxygen partial pressure/fractional inspired oxygen (P/F) ratio of 70. Due to his worsening clinical condition, and young age without multiple co-morbidities, he was transferred to a tertiary center for a higher level of care and consideration of ECMO.

## Discussion

Spontaneous pneumomediastinum (SPM) and spontaneous pneumopericardium (SPP) are less reported entities in COVID-19 pneumonia, and the incidence is unknown. Pneumomediastinum (PM) and pneumopericardium (PP) are rare conditions characterized by the presence of air in the mediastinal and pericardial cavities, respectively. PM can be spontaneous, that occurs without an evident primary cause or secondary, to underlying and predisposing conditions like asthma, bronchiolitis obliterans, illegal drug ingestion, or blunt thoracic trauma [4].

Diffuse alveolar damage (DAD) appears to play a role in the pathogenesis of SPM in SARS patients, and this concept is supported by postmortem studies on lungs of SARS patients revealing extensive features of acute exudative alveolar and vascular injury [5]. PP is typically the result of blunt or penetrating trauma or recent heart surgery. However, in a patient on a ventilator, it may occur by the same mechanism as that which causes most cases of PM, that is, by gas dissecting medially from interstitial emphysema of the lung [6]. Goldman et al. described a case of COVID-19 pneumonia complicated by pneumomediastinum despite the patient not having received mechanical or positive pressure ventilation [7].

SPP unrelated to assisted ventilation is a newly recognized complication of SARS. Belletti et al. conducted an observational study and found the incidence of SPM to be 11.2% and was associated with worse clinical outcomes and increased mortality [8]. Wali et al. reported five cases of PM following intubation in COVID-19 and that the development of PM from the time of tracheal intubation ranged from four hours to 14 days [9]. Possible mechanisms for the development of SPM were increased risk of alveolar damage, tracheobronchial injury, and higher ventilation pressures. The pathophysiology of PM following trauma has well been explained by the Macklin effect, which involves blunt traumatic alveolar rupture, air dissection along broncho vascular sheaths, and subsequent spreading of this blunt pulmonary interstitial emphysema into the mediastinum [10].

During the initial outbreak of COVID-19 in China, extracorporeal membrane oxygenation (ECMO) was utilized for those unresponsive to conventional treatment. Early reports suggested that ECMO has been used in 3% of severe cases with the restoration of adequate oxygenation [11]. Wang et al. described clinical characteristics of 138 COVID-19 patients in Wuhan, China, and reported that 36 patients required ICU care, among which 17 (47%) required mechanical ventilation and four (11.1%) required ECMO [12].

## Conclusions

SPM and PP are increasingly recognized complications of COVID-19 pneumonia and must be suspected when a patient’s condition deteriorates, and appropriate imaging and consultation with cardiothoracic surgery could be critical in managing these complications. Based on our case series, we did note SPM and PP to occur in patients with COVID-19 pneumonia and adversely affect clinical outcomes. SPM is more of a prognostic indicator of worsening COVID-19 infection and is thus associated with a poorer prognosis.
